# Langerhans Cell Histiocytosis Presented as Persistent Diaper Dermatitis: A Case Report

**DOI:** 10.7759/cureus.26606

**Published:** 2022-07-06

**Authors:** Alberto Moscona-Nissan, Guadalupe Maldonado-Colin, Andrea Romo-López, Armando Ventura-Zarate

**Affiliations:** 1 School of Medicine, Universidad Panamericana, Mexico City, MEX; 2 Department of Dermatology, Hospital General Dr. Manuel Gea González, Mexico City, MEX; 3 School of Medicine, Universidad Autónoma del Estado de México, Toluca, MEX

**Keywords:** histiocytes, dermatology, langerhans cells, pediatrics, dermatitis

## Abstract

Langerhans cell histiocytosis presents most frequently in pediatric patients with cutaneous manifestations such as erythematous and scaly papules in the trunk or scalp and macerated plaques in intertriginous sites. We present a case of a seven-month-old patient who was brought with complaints of persistent diaper rash. The patient presented with skin fissures in intertriginous areas and pink color papules dispersed widely in the trunk and perineum. The skin biopsy revealed infiltration of abundant histiocytes, eosinophils, lymphocytes, and plasma cells, being CD207, CD1a, and S-100 positive by immunohistochemistry. Due to the diversified presentations of Langerhans cell histiocytosis and its propensity to mimic other dermatological conditions, physicians should have a clinical suspicion of this disease and consider it as a differential diagnosis among common skin diseases in pediatric patients.

## Introduction

Langerhans cell histiocytosis (LCH) is an idiopathic condition characterized by the accumulation and infiltration of immature myeloid precursors as dendritic cells or macrophages [1]. This disease has features of both inflammatory and neoplastic processes [1]. BRAF mutations in V600E, MAP2K1 mutations, and TP53 alterations have been reported, supporting LCH being a proliferative disorder [2].

LCH occurs predominantly in pediatric patients and presents with various degrees of systemic involvement. The clinical manifestations range from a localized rash to disseminated involvement of bone marrow, lungs, liver, spleen, lymph nodes, gastrointestinal tract, or the pituitary gland [1,3]. Clinicopathological entities of LCH include multifocal multisystem LCH (Letterer-Siwe disease), unifocal or multifocal unisystem affection (eosinophilic granuloma), or pulmonary LCH [4,5].

In infants, LCH presents most frequently with cutaneous manifestations as erythematous and scaly papules in the trunk or scalp, which could mimic seborrheic dermatitis [6]. LCH may also present as macerated or friable plaques in intertriginous sites as axillae [6]. Physicians should assess diverse differential diagnoses such as seborrheic dermatitis, impetigo, intertrigo, atopic dermatitis, candidiasis, and inverse psoriasis when considering LCH, representing a diagnostic challenge. The previous differential diagnoses could cause diaper dermatitis as well [6].

## Case presentation

**Figure 1:**
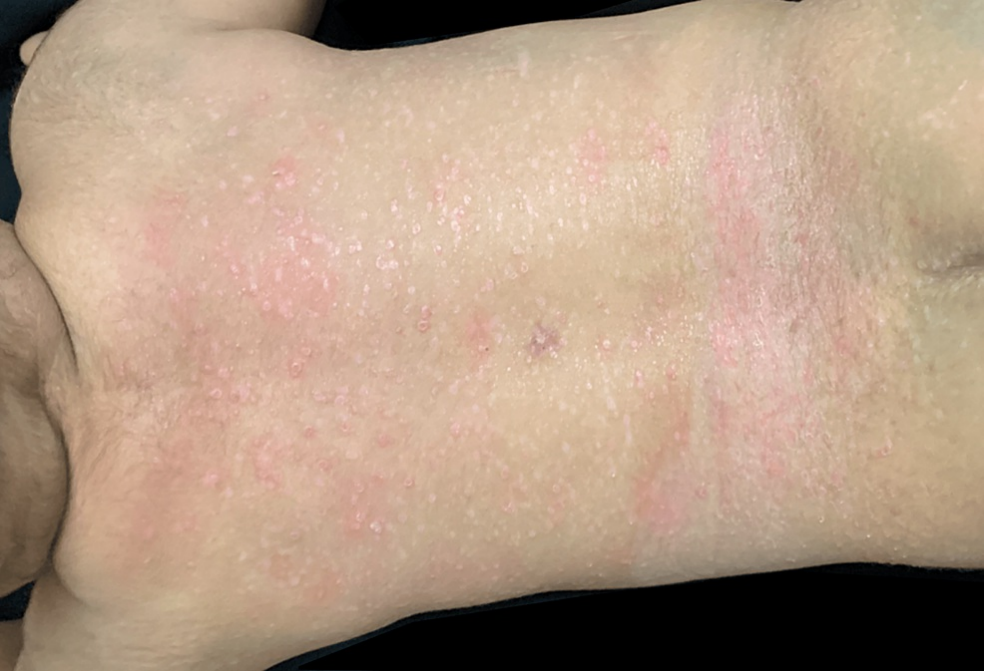
Pink umbilicated papules and scaly erythematous plaque on the back

**Figure 2:**
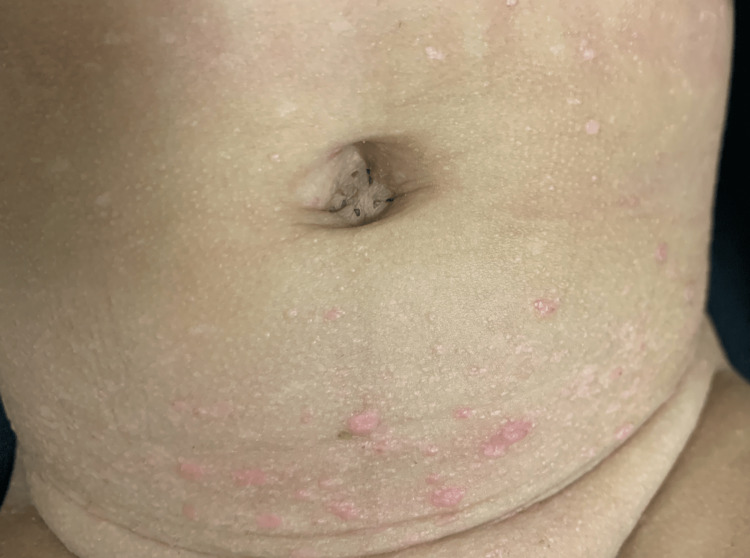
Papules of 1-2 mm on the lower abdomen

A seven-month-old male patient was brought with complaints of diaper rash of three months of evolution with no response to treatment. The patient had no relevant past medical history, and vital signs were found within normal limits. On physical examination, 1-2 mm pink to skin color papules were found disseminated in the trunk, perineum, flexural areas, and axilla (Figures 1, 2).

**Figure 3:**
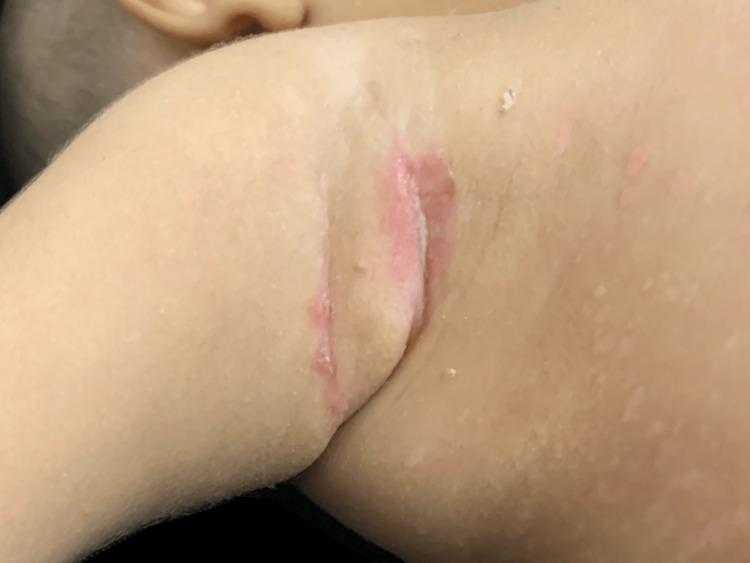
Skin fissure on the right axilla

Additionally, skin fissures were found on the axilla and inguinal region (Figure 3). On the patient's forehead, thin plaques of “flaky and greasy” scales were found, being similar to seborrheic dermatitis. On palpation, neither hepatosplenomegaly nor bone alterations were found.

**Figure 4:**
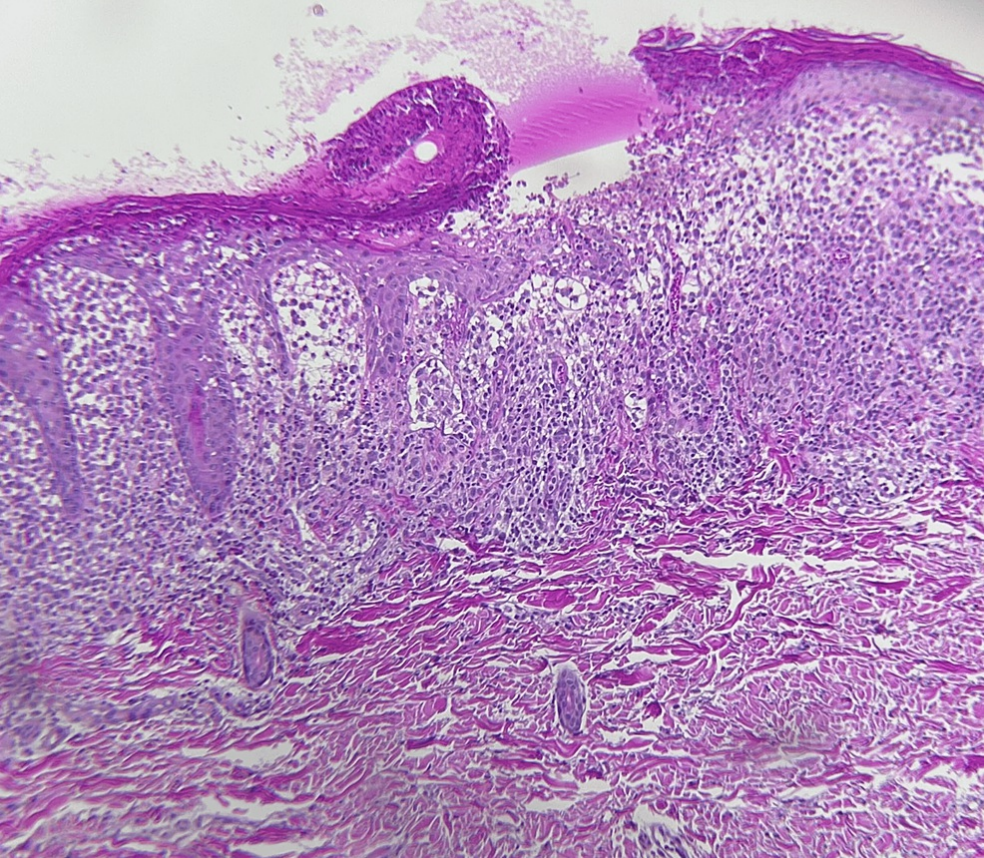
Histological section of the skin showing an ulcerated epidermis and inflammatory cell infiltrate in the dermis and epidermis (hematoxylin and eosin, 20x)

**Figure 5:**
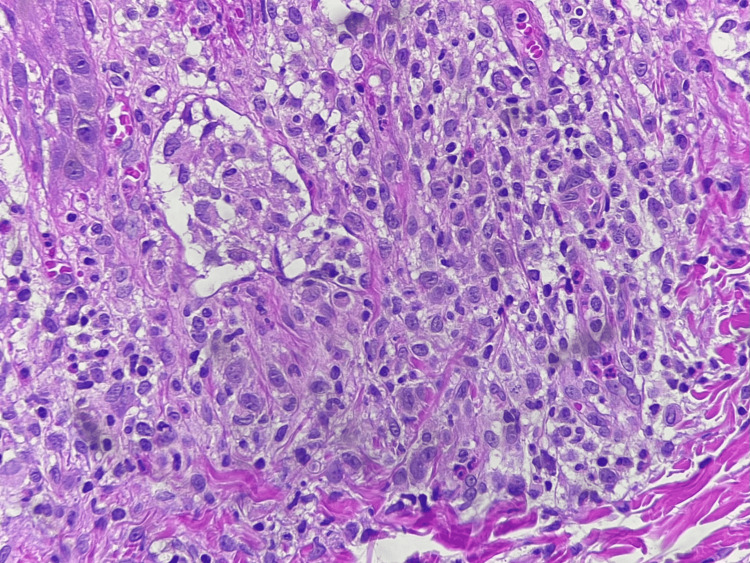
Histological section of the dermis showing infiltration of abundant histiocytes with cytoplasmic vacuolation and microabscesses in the dermis (hematoxylin and eosin, 40x)

An incisional skin biopsy was performed, and microscopic examination showed an ulcerated epidermis with fibrin, cellular debris, parakeratosis, irregular acanthosis, spongiosis, and hydropic degeneration of epidermal basal cells. In the dermis and epidermis, infiltration of abundant polygonal histiocytes with cytoplasmic vacuolation along with eosinophils, lymphocytes, and plasma cells was observed, being CD207, CD1a, and S-100 positive by immunohistochemistry (Figures 4, 5). Additionally, microabscesses were also noted. The histopathologic diagnosis was superficial perivascular dermatitis with a lichenoid pattern and histiocytic infiltration.

## Discussion

The annual reported incidence of LCH is 4.5 cases per million children under 15 years of age, with a male to female ratio of 1.2:1. The estimated median age at diagnosis is 5.9 years. The incidence of LCH among adults is one to two cases per million, though it is probably underdiagnosed in this population [1,7]. Race and ethnic background appear to influence the risk of LCH development. Registry studies in the United States have shown different incidence rates among diverse ethnicities [1,7].

Although the cause of LCH remains unknown, multiple cytokines have been related to this neoplastic and inflammatory process, and other immune cells interact in LCH pathophysiology, considering Langerhans cells' antigen-presenting function. Studies have demonstrated an association between LCH and BRAF and MAP2K1 gene mutations [2,8].

Clinical presentation of LCH may range from single indolent lesions to a disseminated multisystem disease [4]. LCH spectrum includes a multifocal multisystem disease, which usually affects infants, and clinically presents as cutaneous lesions on the trunk and scalp similar to seborrheic dermatitis. In the majority of multifocal multisystem cases, patients present with hepatosplenomegaly, lymphadenopathy, and pulmonary and bone lesions [4,5]. Certain hematologic alterations such as anemia and thrombocytopenia can be found in addition to recurrent infections, including otitis media and mastoiditis [5].

Unifocal or multifocal unisystem LCH is characterized by the proliferation of Langerhans cells admixed with lymphocytes, eosinophils, neutrophils, and plasma cells. These entities may affect the medullary cavities of bones such as the femur, ribs, and skull [5]. The combination of lytic bone lesions in the cranial vault, diabetes insipidus, and exophthalmos corresponds to the classic triad of Hand-Schüller-Christian disease [9]. Finally, pulmonary LCH is considered a proliferative process frequent in smoker adults [5].

Cutaneous manifestations are seen more commonly in LCH affecting children. LCH usually presents as a rash that is frequently misdiagnosed as atopic or seborrheic dermatitis and remains unresponsive to treatment for these disorders [1,6]. LCH cutaneous manifestations may include scaly papules, nodules, or plaques and may vary from a unique lesion to disseminated involvement and may be accompanied by petechiae due to thrombocytopenia, bloody crusting, or indurated nodules [10].

Cutaneous LCH manifestations could mimic diverse dermatological conditions such as diaper dermatitis, juvenile xanthogranulomas, xanthoma disseminatum, seborrheic dermatitis, psoriasis, cutaneous lupus, fungal infections, and atopic and contact dermatitis [11]. The present case addressed a seven-month-old patient with LCH who was brought due to diaper dermatitis for three months with no response to treatment. Diaper dermatitis is defined as an inflammatory reaction of the perineal and perianal skin, being the most frequent skin alteration in young infants. The main causes of diaper dermatitis are related to skin pH alterations, excessive moisture, friction, and irritation due to detergents, urine, or feces [12,13]. Causes of diaper dermatitis include bacterial infections, atopic dermatitis, chronic irritation, mycoses such as *Candida albicans* and *Malassezia*, and seborrheic dermatitis, among others. Its treatment includes skincare, good hygiene, use of topical emollients, low-potency corticosteroids in certain cases, and antifungals or antibiotics in fungal or bacterial infections, respectively [12,13].

The main histopathological findings of LCH include the accumulation of histiocytes, which present an abundant cytoplasm with vacuoles. The immunohistochemical profile of Langerhans cells shows positivity for CD1a, CD207, and S-100. Birbeck granules in electron microscopy are cytoplasmic organelles consisting of pentalaminar tubules with a dilated terminal end having the shape of a “tennis racquet” [4,5].

Treatment of LCH depends on site and grade of involvement. It may vary from observation, corticosteroid application, radiotherapy, chemotherapy, or surgery [1]. Patient prognosis depends on the presentation, organ involvement, and mutations. Children with BRAF mutations in V600E have been associated with high-risk features, permanent injury, poor short-term response to chemotherapy, and LCH being more aggressive and sometimes resistant to treatment [14]. The estimated probability of survival for patients with LCH at five years post-diagnosis is 90-95% [15]. Even though LCH has high cure rates, severe long-term neurological or endocrine complications may affect the quality of life. Children with liver, spleen, or bone marrow affection are classified as high-risk LCH [1]. Referral and long-term follow-up are essential in the management of this disease [1].

## Conclusions

LCH occurs predominantly in pediatric patients and presents frequently with cutaneous manifestations. Clinical suspicion of LCH among physicians is essential for diagnosis, given LCH's diverse forms of presentation and propensity to mimic other dermatological conditions. Physicians should also consider LCH in the differential diagnosis of extremely common dermatological conditions in infants such as seborrheic dermatitis, diaper dermatitis, mycoses, bacterial infections, and intertrigo, among others. The establishment of a clinicopathological correlation is essential for an accurate diagnosis.
